# Eosinophil Peroxidase Activity is a Reliable and Resilient Biomarker for Eosinophilic Chronic Rhinosinusitis

**DOI:** 10.1002/alr.70286

**Published:** 2026-10-06

**Authors:** Christian Caceres, Evan D. Marsh, Kristine A. Smith, Chelsea E. Pollard, Fok Vun Chan, Fatemeh Zarei Haji Abadi, Prince A. B. Minkah, Paul O. Neilsen, Justin R. Savage, Bryan R. McRae, Benjamin L. Witt, Jeremiah A. Alt, Gretchen M. Oakley, Richard R. Orlandi, Abigail Pulsipher

**Affiliations:** ^1^ Department of Otolaryngology–Head and Neck Surgery University of Utah School of Medicine Salt Lake City Utah USA; ^2^ Department of Biomedical Engineering, College of Engineering University of Utah Salt Lake City Utah USA; ^3^ Utah Center for Nanomedicine University of Utah Salt Lake City Utah USA; ^4^ Echelon Biosciences, Inc. Salt Lake City Utah USA; ^5^ Department of Molecular Pharmaceutics College of Pharmacy Salt Lake City Utah USA; ^6^ Lifespan Technologies Salt Lake City Utah USA; ^7^ GlycoMira Therapeutics, Inc. Salt Lake City Utah USA; ^8^ Department of Anatomic Pathology University of Utah School of Medicine Salt Lake City Utah USA

## 1 Introduction

**Key Points**

- Eosinophil peroxidase (EPX) activity shows diagnostic resilience, reliably identifying eosinophilic chronic rhinosinusitis (eCRS) in real‐world practice.
- Swab EPX activity outperforms swab EPX protein as a measure of eCRS inflammation.
- EPX activity reliably identifies eCRS compared with local and systemic cytokine measures.

Chronic rhinosinusitis (CRS) is a heterogeneous inflammatory disease comprising distinct endotypes defined by underlying molecular and immune profiles [1, 2]. Eosinophilic CRS (eCRS) is characterized by type 2 inflammation, sinonasal tissue eosinophilia, comorbid asthma, and nasal polyposis [3]. Patients with eCRS have differing responses to medical therapy and poorer surgical outcomes compared to patients with non‐eCRS [4], making accurate eCRS identification important for treatment planning.

Eosinophil peroxidase (EPX) protein levels in nasal secretions distinguish eCRS from non‐eCRS, irrespective of polyp status, and correlate with interleukin (IL)‐5 and disease severity, supporting EPX as a biomarker for eosinophilic inflammation and anti–IL‐5–targeted therapies [5, 6]. Middle meatal swab EPX activity similarly distinguishes eCRS from non‐eCRS, correlates with disease severity, and predicts postoperative exacerbations [7, 8]. How middle meatal swab‐assessed EPX protein levels and enzymatic activity relate to each other, and to local and systemic cytokine profiles, remains unclear.

This study evaluated EPX protein and activity–obtained with a middle meatal swab–alongside histologic eosinophil counts, sinonasal tissue and plasma cytokine profiles (types 1, 2, and 3), and disease severity measures in patients on continued medical therapy to assess EPX as a viable biomarker for endotyping real‐world CRS.

## 2 Methods

This observational cohort study evaluated relationships between middle meatal swab‐obtained EPX measures, ethmoid tissue and plasma cytokine levels, and disease severity in adult participants (≥18 years) undergoing endoscopic sinus surgery for refractory CRS [3]. Subjects were enrolled after providing written informed consent under a University of Utah IRB‐approved protocol (IRB_00152513) and stratified into eCRS (≥10 eosinophils per high‐power field [HPF]) and non‐eCRS (<10 eosinophils per HPF) via histopathologic eosinophil counts in ethmoid biopsies [3].

Middle meatal swabs, peripheral blood, and anterior ethmoid biopsies were collected, prepared, and analyzed as previously described [7] and detailed in the Supplementary Materials. Swab‐extracted EPX protein was measured by enzyme‐linked immunosorbent assay per manufacturer instructions. Swab‐extracted EPX activity was quantified using a previously reported colorimetric peroxidase assay [7]. Ethmoid tissue and plasma cytokines were measured using a 13‐cytokine multiplex bead‐based assay. Data are reported as individual values with mean ± standard deviation for each group. Group comparisons were performed using two‐tailed Mann–Whitney U tests, and associations were assessed using Spearman's rank correlation coefficient (*ρ*). Statistical significance was defined as *p* < 0.05.

## 3 Results

Of the 48 enrolled participants, 33 had cytokine values within the assay's detectable range and were included in the analysis. Histopathological stratification classified 8 participants as non‐eCRS and 25 subjects as eCRS, of whom 44% had nasal polyps. Demographic and clinical characteristics are reported in Table S1.

Swab EPX activity was significantly higher in participants with eCRS than in non‐eCRS (Figure 1A, *p* = 0.0154), while swab EPX protein levels did not differ significantly between groups (Figure 1B, *p* = 0.397). Both EPX activity (*r_s_* = 0.439; *p* = 0.0106; Figure 1C) and EPX protein levels (*r_s_* = 0.488; *p* = 0.004; Figure 1D) showed moderate positive correlations with tissue eosinophil counts but were not significantly correlated with one another (*r_s_* = 0.157; *p* = 0.384; Figure 1E). EPX measures were also associated with several objective and subjective measures of disease severity (Table S2).

**FIGURE 1:**
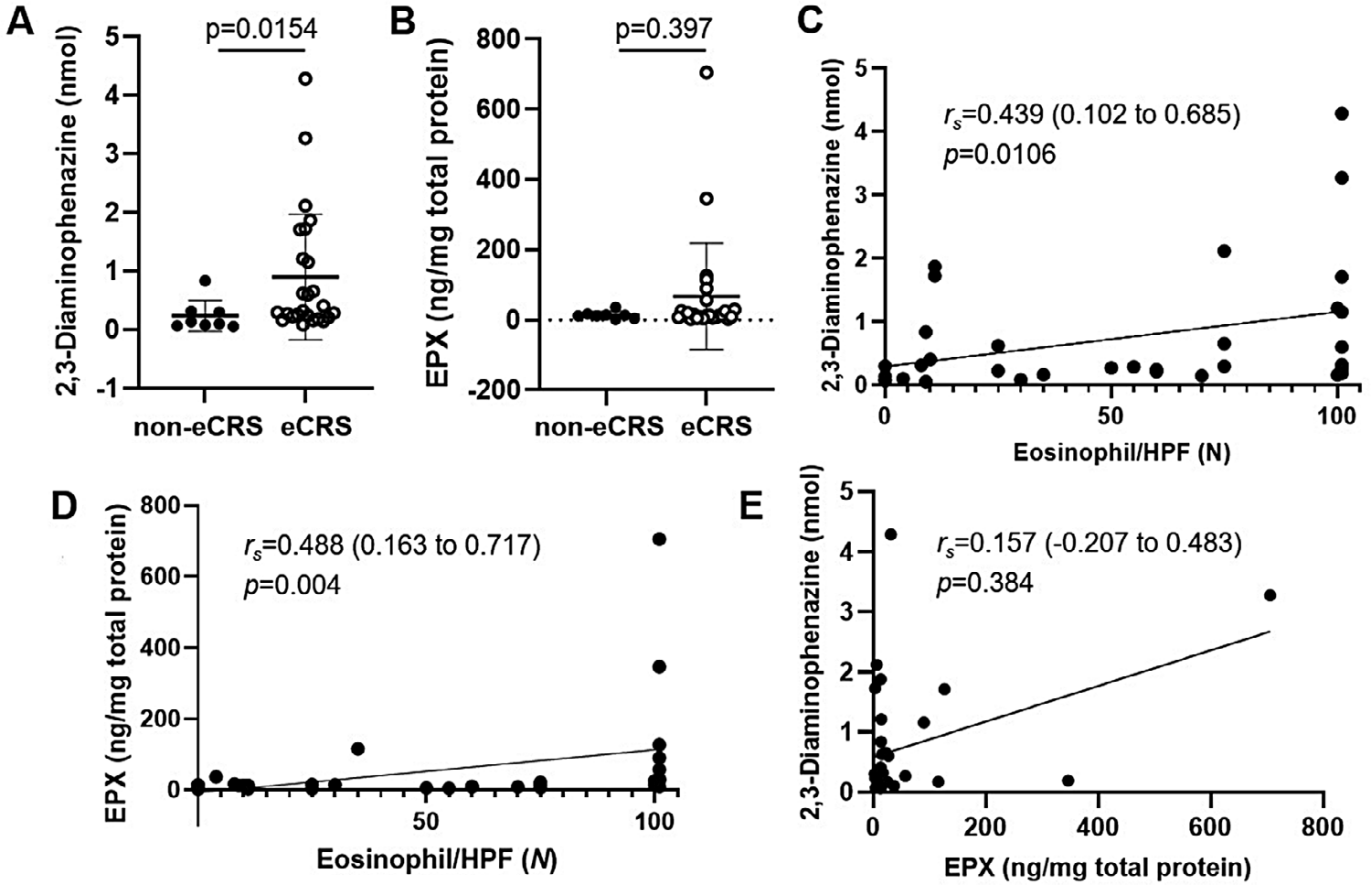
Eosinophil peroxidase (EPX) activity distinguishes eosinophilic chronic rhinosinusitis (eCRS) and correlates with histologic eosinophil counts. (A) Middle meatal swab‐obtained EPX activity is significantly increased in patients with eCRS compared with non‐eCRS (*p* = 0.0154). (B) Middle meatal swab‐obtained EPX protein does not differ significantly between eCRS and non‐eCRS groups (*p* = 0.397). (C) EPX activity demonstrates a moderate positive correlation with histologic eosinophil counts per high‐power field (HPF) (*r_s_* = 0.439, *p* = 0.0106). (D) EPX protein levels also demonstrate a moderate positive correlation with histologic eosinophil counts (*r_s_* = 0.488, *p* = 0.004). (E) EPX protein levels and EPX activity are not significantly correlated with one another (*r_s_* = 0.157, *p* = 0.384). Data are presented as individual values with mean ± standard deviation for group comparisons and with simple linear regression lines for Spearman correlation analyses.

Among the 13 cytokines evaluated in sinonasal tissue homogenates, IL‐2R, IL‐8, IL‐13, and IL‐6 were detectable within the assay range; none differed significantly between eCRS and non‐eCRS (Figure 2A–D). Of the 13 cytokines evaluated in plasma, IL‐2R, IL‐8, interferon‐γ, IL‐13, IL‐6, and IL‐10 were detectable; none differed significantly between groups (Figure 2E–J). Several sinonasal tissue cytokines correlated with eosinophil counts and EPX activity (Table S3), whereas plasma cytokines correlated strongly with one another (Table S4).

**FIGURE 2:**
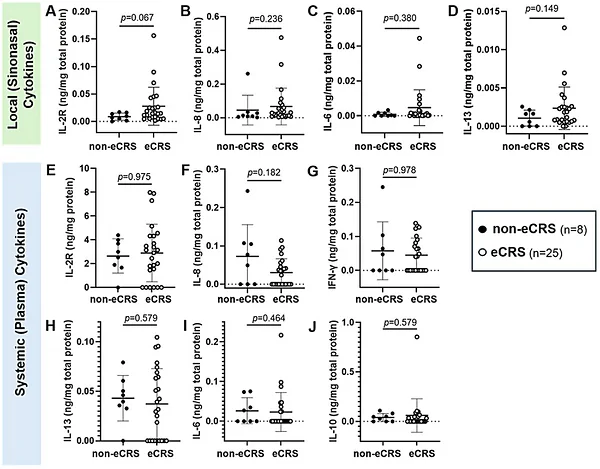
Sinonasal tissue and plasma cytokines were not significantly different between eosinophilic chronic rhinosinusitis (eCRS) and non‐eCRS. Among the 13 cytokines evaluated in sinonasal tissues, interleukin (IL)‐2R, IL‐8, IL‐6, and IL‐13 were detectable within the assay range. Local (A) IL‐2R (*p* = 0.0670), (B) IL‐8 (*p* = 0.236), (C) IL‐6 (*p* = 0.380), and (D) IL‐13 (*p* = 0.149) were not significantly different between groups. Among the 13 cytokines evaluated in plasma samples, IL‐2R, IL‐8, interferon (IFN)‐γ, IL‐13, IL‐6, and IL‐10 were detectable within the assay range. No significant differences in systemic (E) IL‐2R (*p* = 0.975), (F) IL‐8 (*p* = 0.182), (G) IFN‐γ (*p* = 0.978), (H) IL‐13 (*p* = 0.579), (I) IL‐6 (*p* = 0.464), and (J) IL‐10 (*p* = 0.579) were demonstrated between eCRS and non‐eCRS. Data are presented as individual values with mean ± standard deviation for group comparisons.

## 4 Discussion

Middle meatal swab EPX activity distinguished eCRS from non‐eCRS (Figure 1A), whereas middle meatal swab EPX protein levels and local and systemic cytokines did not significantly differ between groups. Both EPX activity and protein correlated with histologic eosinophil counts (Figure 1C,D) yet were not correlated with each other (Figure 1E), suggesting EPX activity and protein reflect unique facets of eosinophilic inflammation.

Local tissue cytokines, IL‐2R, IL‐8, and IL‐13, correlated with eosinophil burden, and IL‐2R and IL‐6 correlated with EPX activity (Table S3). EPX activity showed associations with several local cytokines compared to EPX protein (Table S3), suggesting that local cytokine signaling reflects eosinophil activation.

Systemic plasma cytokines did not distinguish eCRS status and were not associated with local eosinophilic markers; however, there were correlations between different systemic plasma cytokines (Table S4). These results are consistent with coordinated systemic immune activity in patients with CRS, independent of sinonasal inflammation. These findings emphasize the need for clinically relevant local biomarkers of eosinophilic disease, such as minimally invasive EPX‐based measures [5, 9].

Participants continued intranasal corticosteroid therapy preoperatively, which likely accounts for the absence of elevated IL‐4, IL‐5, and IL‐13, despite differences in eosinophil burden and disease severity. EPX activity remained diagnostic under these conditions, strengthening its use as a biomarker for real‐world CRS populations, where treatment interruption before sampling is impractical [8]. Because EPX activity can be measured from swabs using a simple colorimetric assay, these findings support integrating EPX activity assessment into routine CRS endotyping, alongside clinical evaluation and imaging, to guide decisions on corticosteroid and biologic therapy.

This study is limited by a modest sample size, group imbalance, and a cross‐sectional design, which precluded assessing temporal or treatment‐response dynamics. Larger longitudinal, multicenter studies are needed to validate EPX activity as a point‐of‐care biomarker for CRS endotyping [10].

In summary, middle meatal swab EPX activity is a reliable and clinically resilient biomarker for identifying eCRS under real‐world clinical conditions compared to EPX protein and local or systemic cytokines.

## Funding

This work was funded by a grant award from the National Institute of Allergy and Infectious Diseases under award number R41AI179525. Research reported in this publication was supported by the National Center for Advancing Translational Sciences of the National Institutes of Health under Award Numbers UM1TR004409 and K12TR004413. The content is solely the responsibility of the authors and does not necessarily represent the official views of the National Institutes of Health.

## Conflicts of Interest

**Abigail Pulsipher**, **Justin R. Savage**, and **Jeremiah A. Alt** have a financial interest in GlycoMira Therapeutics. **Abigail Pulsipher**, **Kristine A. Smith**, and **Richard R. Orlandi** have a financial interest in EoSight Diagnostics, Inc. **Jeremiah A. Alt** is a consultant speaker for Sanofi and GlaxoSmithKline and is an opinion leader for Medtronic. The remaining authors declare no conflicts of interest.

## Supporting information




**Supporting File1**: alr70286‐sup‐0001‐SupMat.docx.
