# Supplementary material for: Eosinophil Peroxidase Activity is a Reliable and Resilient Biomarker for Eosinophilic Chronic Rhinosinusitis
Source: Int Forum Allergy Rhinol. 2026 Oct 6;16(11):1377–80. doi: 10.1002/alr.70286 (PMC13731147; doi:10.1002/alr.70286)
Supplement: Supplementary file 1 [file ALR-16-1377-s001.docx]

**Supplementary Materials**

**Methods**

**Study participants and data collection**

A prospective observational cohort study was conducted to evaluate relationships between eosinophil peroxidase (EPX) measures, cytokine profiles, disease severity, and demographics in participants diagnosed with refractory CRS.^1^ Adult participants (≥18 years) undergoing endoscopic sinus surgery (ESS) were enrolled after providing written informed consent under protocols approved by the University of Utah Institutional Review Board (IRB_00152513).

Subjects were stratified based on histopathologic eosinophil counts in anterior ethmoid tissue biopsies into eCRS (eCRS; ≥10 eosinophils per high-power field [HPF]) and non-eCRS (non-eCRS; <10 eosinophils per HPF) in accordance with the European Position Paper on Rhinosinusitis and Nasal Polyps 2020.^2^ Subjects receiving oral corticosteroids within four weeks prior to surgery and/or biologic therapies and those with cystic fibrosis, granulomatosis with polyangiitis, myelodysplastic syndrome, or recurrent acute rhinosinusitis were excluded. Subjects undergoing intranasal corticosteroids were included.

All participants underwent a standardized clinical evaluation, including medical history, head and neck examination, and computed tomography (CT) imaging, and were scored using the Lund–Mackay system (range, 0–24). Endoscopy was performed and scored using the Lund-Kennedy system (range, 0-12). Patient-reported symptom burden was assessed using the 22-item Sino-Nasal Outcome Test (SNOT-22; range, 0–110). Demographic information (age, sex, self-reported race and ethnicity) and comorbidities were recorded.

**Histopathologic eosinophil quantification**

Ethmoid tissue biopsies were fixed, paraffin-embedded, sectioned (4-µm-thick), and stained with hematoxylin and eosin at the ARUP Research Histology Core at the Huntsman Cancer Institute. Histology slides were reviewed by a board-certified head and neck pathologist blinded to clinical and biomarker data. Intact eosinophils were counted at 400x and reported as count per high-power field (HPF).

**Middle meatal swab collection**

Middle meatal swabs were collected intraoperatively, prior to ESS, under endoscopic visualization, allowing direct comparison with ethmoid tissue biopsy-obtained eosinophil counts obtained at the same time. Two swabs were obtained from each participant by sampling the epithelial surface of the left and right middle meatus. Swabs were flash frozen on dry ice and stored at −80°C until analysis.

**Peripheral blood collection and plasma preparation**

Up to 8 mL of peripheral blood was collected into EDTA or citrate-coated vacutainer tubes (BD, Logan, UT) from each participant at the time of preoperative intravenous placement. Blood was centrifuged for 10 minutes at 2,500 rpm, and the plasma fraction was separated, aliquoted, and stored at −80°C until analysis.

**Ethmoid tissue collection and homogenization**

Anterior ethmoid tissue biopsies were obtained intraoperatively (~1-2 mm^2^) when clinically indicated, flash frozen, and stored at −80°C. Tissues were homogenized in phosphate-buffered saline containing protease inhibitors (Promega, WI, USA) and glycerol (20% v/v), then centrifuged (1,200 rpm) to obtain the supernatants. Total protein concentration was determined using the Bradford protein assay.^3^

**Middle meatal swab extraction**

Middle meatal swabs were extracted by adding 0.5 mL of 0.4% Triton X-100 in water to each swab (kept on ice), followed by vortexing (5 × 3 s with 15 s rest intervals on ice). Swabs were then centrifuged (10,000 rpm, 2 min, 4°C), and the supernatant was collected for analysis. Extracts were aliquoted and stored at −80°C until assay.

**EPX enzyme activity measurements**

EPX activity was quantified using a previously reported^4^ colorimetric peroxidase assay modified for 96-well plate format. Swab extracts were analyzed using a kinetic screen in a reaction mixture containing 5 mM o-phenylenediamine substrate and 0.03% v/v hydrogen peroxide in 1 M Tris-HCl buffer (pH 8) for 30 minutes at room temperature. Reactions were quenched with 4 M sulfuric acid at 30 minutes, and relative absorbance units (R.A.U.) were measured at 492 nm using a spectrophotometer reader. Raw R.A.U. were compared against a standard curve of 2,3-diaminophenazine–o-phenylenediamine-converted enzymatic product–and reported as averaged values (nmol) ± standard deviation across left and right middle meatal swabs for each group. Analyte detection ranged from 11.25-180 ng/mL. Samples visibly contaminated with blood or mucopurulent material were excluded from analysis. All chemicals and buffers were obtained from Sigma Aldrich (St. Louis, MO, USA).

**EPX protein quantification**

Swab extracts prepared for the EPX activity assay were also evaluated for EPX protein concentration using a commercially available EPX enzyme-linked immunosorbent assay kit (MyBioSource, CA, USA). EPX values were normalized to total swab extract protein concentration using the Bradford Assay per manufacturer instructions.^3^ Analyte detection ranged from 1.25-80 ng/mL. EPX protein is reported as nanograms (ng) of EPX per milligram (mg) of total protein ± standard deviation for each group.

**Cytokine quantification in ethmoid tissue homogenates and plasma samples**

Ethmoid tissue homogenates and peripheral blood plasma samples were submitted to ARUP Laboratories (Salt Lake City, UT) for cytokine quantification using a multiplex bead-based immunoassay (Cytokine Panel, 13). The cytokine panel included the following: pro-inflammatory type 1-associated interleukin (IL)-2R, IL-2, IL-12, IL-8, tumor necrosis factor (TNF)-α, and interferon gamma (IFN-γ); type 2-associated IL-4, IL-5, and IL-13; type 3-associated IL-17; pleiotropic IL-6; and anti-inflammatory IL-10. Analyte detection ranged from 1.4 pg/mL to 400 Pg/mL for all cytokines except IL-2R (4.7 pg/mL to 1.2 Mg/mL). Cytokine concentrations were normalized to total protein concentration in tissue homogenates or plasma determined by the Bradford Assay^3^ and expressed as ng of cytokine per mg of total protein ± standard deviation for each group. All assays were performed according to manufacturer specifications and laboratory quality control standards at ARUP (Salt Lake City, UT).

**Statistical analysis**

Group comparisons between eCRS and non-eCRS were performed using two-tailed Mann–Whitney U tests. Associations between continuous variables were assessed using Spearman’s rank correlation coefficient (ρ). Exact p-values are reported where available; p<0.05 was considered statistically significant. Sample sizes varied slightly across analyses due to missing clinical data: Lund–Mackay CT scores were available for 30 participants, Lund–Kennedy endoscopy scores for 32, and all other analyses included 33 participants. Statistical analyses were performed using GraphPad Prism (version 10.6.0).

**Supplemental Tables**

**Supplemental Table 1.** Patient Demographics.

| Characteristic | Non-eCRS (n=8) | eCRS (n=25) | *p*-value |
| --- | --- | --- | --- |
| Sex, n (%) | 8 (24) | 25 (76) | 0.917 |
| Male | 5 (62) | 13 (52) |  |
| Female | 3 (38) | 12 (48) |  |
| Age, mean ± SD | 59.75 ± 12.60 | 42.64 ± 15.98 | 0.0168 |
| Ethnicity,* n (%) |  |  | 1.00 |
| Hispanic | 0 (0) | 1 (4) |  |
| Non-Hispanic | 8 (100) | 23 (92) |  |
| Undisclosed | 0 (0) | 1 (4) |  |
| Polyp status, n (%) |  |  | 0.0109 |
| Yes | 0 (0) | 11 (44) |  |
| No | 8 (100) | 14 (56) |  |
| Comorbidities, n (%) |  |  |  |
| Allergic rhinitis | 3 (38) | 10 (40) | 1.00 |
| Allergies* | 6 (75) | 20 (80) | 1.00 |
| Asthma | 0 (0) | 13 (52) | 0.018 |
| Migraine | 2 (25) | 6 (24) | 1.00 |
| Diabetes | 1 (12) | 4 (16) | 0.789 |
| GERD | 4 (50) | 3 (12) | 0.083 |
| COPD | 1 (12) | 0 (0) | 0.580 |
| OSA | 4 (50) | 8 (32) | 0.605 |
| Clinical Metrics, mean ± SD | | | |
| Lund-Mackay CT Score | 6.3 ± 3.8 | 14.1 ± 5.1 | 0.0002 |
| Lund-Kennedy Score | 2.14 ± 1.68 | 4.12 ± 2.85 | 0.082 |
| SNOT-22 score, mean ± SD | 37.0 ± 13.3 | 48.6 ± 20.7 | 0.216 |

CT: computed tomography; GERD: gastroesophageal reflux disease; COPD: chronic obstructive pulmonary disease; SNOT: sinonasal outcomes test, OSA: obstructive sleep apnea. *patient-reported

**Supplemental Table 2.** Spearman correlations between swab-collected EPX measures and CRS disease severity.

| Comparison |  | Rho (95% CI) | P value |
| --- | --- | --- | --- |
| EPX vs. SNOT-22 (n=33) | | 0.495 (0.173 to 0.722) | 0.003 |
| EPX vs. *Lund-Mackay Score (n=30) | | 0.441 (0.085 to 0.697) | 0.015 |
| EPX vs. ^#^Lund-Kennedy Score (n=32) | | 0.063 (-0.302 to 0.412) | 0.731 |
| EPX Activity vs. SNOT-22 (n=33) | | 0.305 (-0.054 to 0.594) | 0.085 |
| EPX Activity vs. *Lund-Mackay Score (n=30) | | 0.443 (0.088 to 0.699) | 0.014 |
| EPX Activity vs. ^#^Lund-Kennedy Score (n=32) | | 0.516 (0.194 to 0.738) | 0.002 |

*computed tomography scoring system, ^#^endoscopic scoring system, EPX: eosinophil peroxidase, SNOT: sinonasal outcomes test

| Supplemental Table 3. Spearman correlations between sinonasal eosinophil counts, EPX measures, and tissue cytokines (n=33). | | | | |
| --- | --- | --- | --- | --- |
| Variable | **IL-2R** | **IL-6** | **IL-8** | **IL-13** |
| Eosinophil Count | 0.456** | 0.306 | 0.360* | 0.374* |
| EPX Activity | 0.437* | 0.410* | 0.223 | 0.187 |
| EPX | 0.210 | 0.343 | 0.039 | 0.047 |
| IL-2R | — | 0.458** | 0.700**** | 0.736**** |
| IL-6 | 0.458** | — | 0.581*** | 0.268 |
| IL-8 | 0.700**** | 0.581*** | — | 0.580*** |
| IL-13 | 0.736**** | 0.268 | 0.580*** | — |

Values represent Spearman correlation coefficients (*r_s_*); *p<*0.05 (*), *p*<0.01 (**), *p*<0.001 (***), and *p*<0.0001 (****)

| Supplemental Table 4. Spearman correlations between sinonasal eosinophil counts and EPX measures and systemic cytokines (n=33) measured in plasma. | | | | | | |
| --- | --- | --- | --- | --- | --- | --- |
| Variable | **IL-2R** | **IL-6** | **IL-8** | **IL-10** | **IL-13** | **IFN-γ** |
| Eosinophil Count | -0.055 | -0.138 | -0.189 | -0.160 | -0.158 | -0.234 |
| EPX Activity | -0.159 | 0.152 | -0.080 | -0.129 | -0.062 | -0.067 |
| EPX | -0.341 | -0.406* | -0.431* | -0.553*** | -0.318 | -0.594*** |
| IL-2R | — | 0.212 | 0.454** | 0.550*** | 0.628**** | 0.567*** |
| IL-6 | 0.212 | — | 0.506** | 0.528** | 0.373* | 0.462** |
| IL-8 | 0.454** | 0.506** | — | 0.651**** | 0.599*** | 0.386* |
| IL-10 | 0.550*** | 0.528** | 0.651**** | — | 0.601*** | 0.450** |
| IL-13 | 0.628**** | 0.373* | 0.599*** | 0.601*** | — | 0.454** |
| IFN-γ | 0.567*** | 0.462** | 0.386* | 0.450** | 0.454** | — |

Values represent Spearman correlation coefficients (*r_s_*); *p<*0.05 (*), *p*<0.01 (**), *p*<0.001 (***), and *p*<0.0001 (****)

**References**

1.	Rosenfeld RM, Piccirillo JF, Chandrasekhar SS, Brook I, Ashok Kumar K, Kramper M, et al. Clinical Practice Guideline (Update): Adult Sinusitis. *Otolaryngol Head Neck Surg*. 2015;152(S2):S1–S39.

2.	Fokkens WJ, Lund VJ, Hopkins C, Hellings PW, Kern R, Reitsma S, et al. European Position Paper on Rhinosinusitis and Nasal Polyps 2020. *Rhinology*. 2020;58(Suppl S29):1–464.

3.	Bradford MM. A rapid and sensitive method for the quantitation of microgram quantities of protein utilizing the principle of protein-dye binding. *Anal Biochem*. 1976;72(1):248–54.

4.	Smith KA, Gill AS, Pollard CE, Sumsion JS, Saffari H, Ashby S, et al. An eosinophil peroxidase activity assay accurately predicts eosinophilic chronic rhinosinusitis. Journal of Allergy and Clinical Immunology. 2023;152(2):400–7.
